# Unlocking economic gains: the impact of image-guided brachytherapy on cervical cancer treatment in Thailand

**DOI:** 10.3389/fpubh.2025.1725415

**Published:** 2026-02-02

**Authors:** Ekkasit Tharavichitkul, Imjai Chitapanarux, Patumrat Sripan, Chokaew Tovanabutra, Temsak Phungrassami, Rungarun Kittichet, Chawalit Lakdee, Tussawan Asakit, Kanokpis Towanamchai, Sirinthip Songwutwichai, Komsan Thamronganantasakul, Tharatorn Tungkasamit, Attapol Pinitpatcharalert, Sutthisak Kulpisitthicharoen, Somying Wongsrita, Rachata Banlengchit, Darat Khamchompoo, Eduardo Zubizarreta, Yavuz Anacak

**Affiliations:** 1 Division of Radiation Oncology, Department of Radiology, Faculty of Medicine, Chiang Mai University, Chiang Mai, Thailand; 2 Research Institute for Health Sciences, Chiang Mai University, Chiang Mai, Thailand; 3 Radiation Oncology Section, Chonburi Cancer Hospital, Chonburi, Thailand; 4 Division of Radiation Oncology, Department of Radiology, Faculty of Medicine, Prince of Songkhla University, Songkhla, Thailand; 5 Division of Radiotherapy, Department of Radiology, Buddhachinaraj Hospital, Phitsanulok, Thailand; 6 Radiation Oncology Section, Lampang Cancer Hospital, Lampang, Thailand; 7 Radiation Oncology Division, Bhumibol Adulyadej Hospital, Directorate of Medical Services Royal Thai Air Force, Bangkok, Thailand; 8 Radiation Oncology Department, Phramongkutklao Hospital, Bangkok, Thailand; 9 Division of Radiation Oncology, Department of Radiology, Faculty of Medicine, Khon Kaen University, Khon Kaen, Thailand; 10 National Cancer Institute, Department of Medical Services, Ministry of Public Health, Bangkok, Thailand; 11 Division of Radiation Oncology, Department of Radiology, Faculty of Medicine, Thammasat University, Pathum Thani, Thailand; 12 Department of Radiotherapy, Lopburi Cancer Hospital, Lopburi, Thailand; 13 Division of Radiation Oncology, Maharaj Nakornratchasima Hospital, Nakornratchasima, Thailand; 14 Division of Radiotherapy, Department of Radiology, Ratchaburi Hospital, Ratchaburi, Thailand; 15 Nakornping Cancer Center, Nakornping Hospital, Chiang Mai, Thailand; 16 International Atomic Energy Agency, Vienna, Austria; 17 Department of Radiation Oncology, Ege University Faculty of Medicine & Hospital, Izmir, Türkiye

**Keywords:** cervical cancer, economic benefits, image-guided brachytherapy, surveys, utilization

## Abstract

**Objective:**

This study aims to bridge the existing gap in knowledge by assessing the financial impact of image-guided brachytherapy (IGBT) in the management of cervical cancer in Thailand.

**Methods:**

A web-based questionnaire was developed in 2019 to collect data from 14 radiotherapy centers across all regions of Thailand. The survey gathered information on the use of brachytherapy for cervical cancer treatment, encompassing both conventional brachytherapy (point-based prescription) and IGBT (volume-based prescription). Data on infrastructure, workforce, and costs were also collected, and predictions for radiotherapy usage in cervical cancer were calculated. The actual image-guided brachytherapy utilization (A-IGBTU) rate was calculated by dividing the IGBT fractions by the total brachytherapy fractions and multiplying the result by 100. The Radiotherapy Resources and Cost Calculator (RRCC; version 21.0) was used to assess shortages, while the economic model was based on clinical outcomes and toxicity models.

**Results:**

Our survey revealed that 18,024 new patients were treated with radiotherapy, including 2,950 patients with gynecological cancers. Among these, cervical cancer accounted for 72% of the cases. The actual utilization rate of IGBT for cervical cancer was 33%. The RRCC (version 21.0) highlighted workforce shortages for radiation oncologists (ROs), medical physicists (MPs), and radiation technologists (RTTs) at 42, 63, and 61%, respectively. In the clinical outcome model, IGBT generated a total income of USD1,492,563. In the toxicity model, IGBT reduced the costs associated with treating grade-3 and grade-4 toxicities by at least 50%.

**Conclusion:**

The actual utilization rate of IGBT for treating cervical cancer patients was 33%. The RRCC (version 21.0) highlighted workforce shortages across all roles. In our analysis, IGBT generated higher total income and significantly reduced the costs associated with treating severe toxicities.

## Introduction

According to the data obtained from the Thailand National Cancer Institute, in 2021, cervical cancer was the second most common cancer among women in Thailand after breast cancer, with an estimated 13.8% of new cases (1). The highest incidence was reported in Central and Eastern Thailand, with an age-standardized incidence rate (ASR) of 14.4 cases per 100,000 women per year, followed by the North (ASR of 13.4), Northeast (ASR of 9.5), and South Thailand (ASR of 8.4) (1, 2). Previous studies conducted in Bangkok and the Northeast region of Thailand revealed that locally advanced cervical cancer (LACC) had the highest ratio among all cervical cancer stages (3, 4).

Brachytherapy (BT) is an important component of treatment for patients with LACC. Numerous studies reported that image-guided brachytherapy (IGBT) enhances local control and reduces treatment-related complications (5–7).

Image-guided brachytherapy (IGBT), facilitated by magnetic resonance imaging (MRI) or computed tomography (CT), offers significant dosimetric advantages. The primary benefit of this therapy is its ability to increase the cumulative radiation dose delivered to the target volume while simultaneously limiting the dose to adjacent organs at risk (OARs). This precision is directly correlated with an improvement in clinical outcomes (8, 9). Consequently, in Thailand, IGBT using CT or MRI guidance has been widely adopted across all medical universities and select specialized radiotherapy centers.

A cost-effectiveness analysis comparing IGBT and two-dimensional (2D) brachytherapy for treating LACC was conducted in the United States, a high-income country, and demonstrated that IGBT for LACC was more cost-effective than 2D conventional brachytherapy (10).

To our knowledge, no published studies on IGBT have examined the medico-economic impact of this treatment. Following the publication of the first positive clinical results of IGBT globally, radiotherapy departments at university hospitals in Thailand began implementing this treatment method. In this study, we assessed the utilization rate of IGBT and explored the economic impact of this treatment modality in cervical cancer.

## Materials and methods

Ethical approval for this study was exempted by the Institutional Review Board, Faculty of Medicine, Chiang Mai University (Reference No. Exemption-9198/2022). The study was conducted in accordance with the Declaration of Helsinki and relevant institutional guidelines.

### Step 1.1: web-based questionnaire and data collection

A web-based questionnaire was developed in 2019 to gather data from 14 radiotherapy centers, including Chiang Mai University, Nakornping Hospital, Lampang Cancer Hospital, Udonthani Cancer Hospital, Khonkaen University, Nakornrachasima Hospital, Bhuthachinnaraj Hospital, Prince of Songkhla University, Rachaburi Hospital, Chonburi Cancer Hospital, Lopburi Cancer Hospital, Bhumibol Adulyadej Hospital, Phramongkutklao Hospital, and Thammasat University. The questionnaire was used for collecting information on equipment, personnel, the number of cervical cancer cases, and treatment specifics (see Supplementary material).

Data regarding the use of brachytherapy for cervical cancer were collected from 14 centers, focusing on both two-dimensional brachytherapy (2DBT; point A prescription) and IGBT (volume-based prescription). In a meta-analysis by Hande et al. (11), the optimal utilization rate of image-guided brachytherapy (O-IGBTU) was defined as 100%, highlighting the therapy’s benefits in achieving 3-year local control and disease-free survival rates.

The actual image-guided brachytherapy utilization (A-IGBTU) rate was calculated by dividing the number of IGBT fractions by the total number of brachytherapy fractions and then multiplying the result by 100.


$$\mathrm{Actual}-\mathrm{IGBT rate}=\left(\mathrm{Fractions of}\frac{\mathrm{IGBT}}{\mathrm{Total}\;\mathrm{BT}}\right)\times 100$$

### Step 1.2: prediction of utilization of RT for cervical cancers in Thailand

Multiple sources of data were used for predicting actual radiation therapy (RT) utilization rate for cervical cancers in Thailand: the number of cervical cancer cases are collected from Cancer in Thailand report volume VIII–X covering 2012–2018, in combination with estimated number of cervical cancer cases from GLOBOCAN from 2019 to 2040, and the data on cervical cancers from the Thai Association of Radiation Oncology (THASTRO) from the THASTRO website.^1^

The details pertinent to cervical cancer cases treated by radiotherapy and brachytherapy were collected from the centers. A negative binomial regression model was used to predict the number of radiotherapy requirements for gynecological (GYN) and cervical cancer by 2040.

First, the number of national GYN cancers was predicted through 2040 using temporal changes, based on data from the Cancer in Thailand report and a negative binomial regression model. Then, using the same model, the requirements for radiotherapy for GYN cancers were predicted, considering variables such as time and the number of GYN cancer cases.

### Step 2: calculation of infrastructure shortage

In this step, we used data on personnel, machines, staff salaries, and the number of patients treated with radiotherapy in 2019, collected from the 14 centers. These data were entered into the Radiotherapy Resources and Cost Calculator (RRCC) version 21.0 (12). Then, the details of the shortage of workforce and machines were evaluated (see Supplementary material).

### Step 3: calculation of loss and benefit

#### Step 3.1: clinical outcome model developments

In this step, the A-IGBTU data collected in Step 1 were utilized for further analysis. Clinical outcomes of brachytherapy, including both conventional and IGBT, were collected from the literature to assess overall survival. The “Percentage of Difference in Overall Survival Rate” was calculated by comparing the overall survival rates between 2DBT (point A) and IGBT (volume), as reported in the studies by Lorvidhaya et al. and Tharavichitkul et al. (13, 14). We adapted the framework established by the Global Task Force on Radiotherapy for Cancer Control (GTFRCC) (15) as shown in Supplementary Diagram 1 to estimate the health and economic impact of expanding access to radiotherapy for cervical cancer in low to upper-middle-income countries.

The “patient life-years,” expressed in years, for surviving patients, were calculated from the year of treatment to the year of death. The “total income of surviving patients” expressed in Thai currency (Thai Baht [THB]) was calculated by multiplying the average annual income of Thai people in THB by the number of surviving patients until retirement.

#### Step 3.2: toxicity model developments

We retrospectively obtained data on radiation-induced toxicities from cervical cancer treatment from the Medical Record Unit of the Faculty of Medicine, Chiang Mai University. We searched for details of “cervical cancer” cases with “proctitis,” “cystitis,” “hematuria,” “hematochezia,” “bowel obstruction,” or “fistulas” from 2013 to 2023 in terms of “episodes.” These data were then cross-referenced with our RT medical records. Episodes that could not be traced back to our patients in the RT medical records were excluded, as well as episodes in patients who received only external beam RT.

We gathered and analyzed episodes in patients who underwent brachytherapy, which was classified as either two-dimensional brachytherapy (2DBT) or IGBT. The severity of each episode was rated as grades 1–4 in accordance with the Common Terminology Criteria for Adverse Events (CTCAE, version 5.0) (16) for 2DBT or three-dimensional brachytherapy (3DBT). To determine the “number of patients,” we documented the episodes with the highest toxicity grade for each patient. We evaluated the number of patients who experienced toxicities associated with each BT technique.

### Data analysis

All data for each step were analyzed collectively to address the project’s objectives. Descriptive data were processed using basic calculations in Microsoft Excel. For more complex data, including predictive analyses, STATA version 16.0 was employed in step 1.2. To determine facility shortages from the 14 centers, RRCC version 21.0 was used in step 2 (12).

## Results

### Step 1.1: facility of 14 RT centers

**Figure 1:**
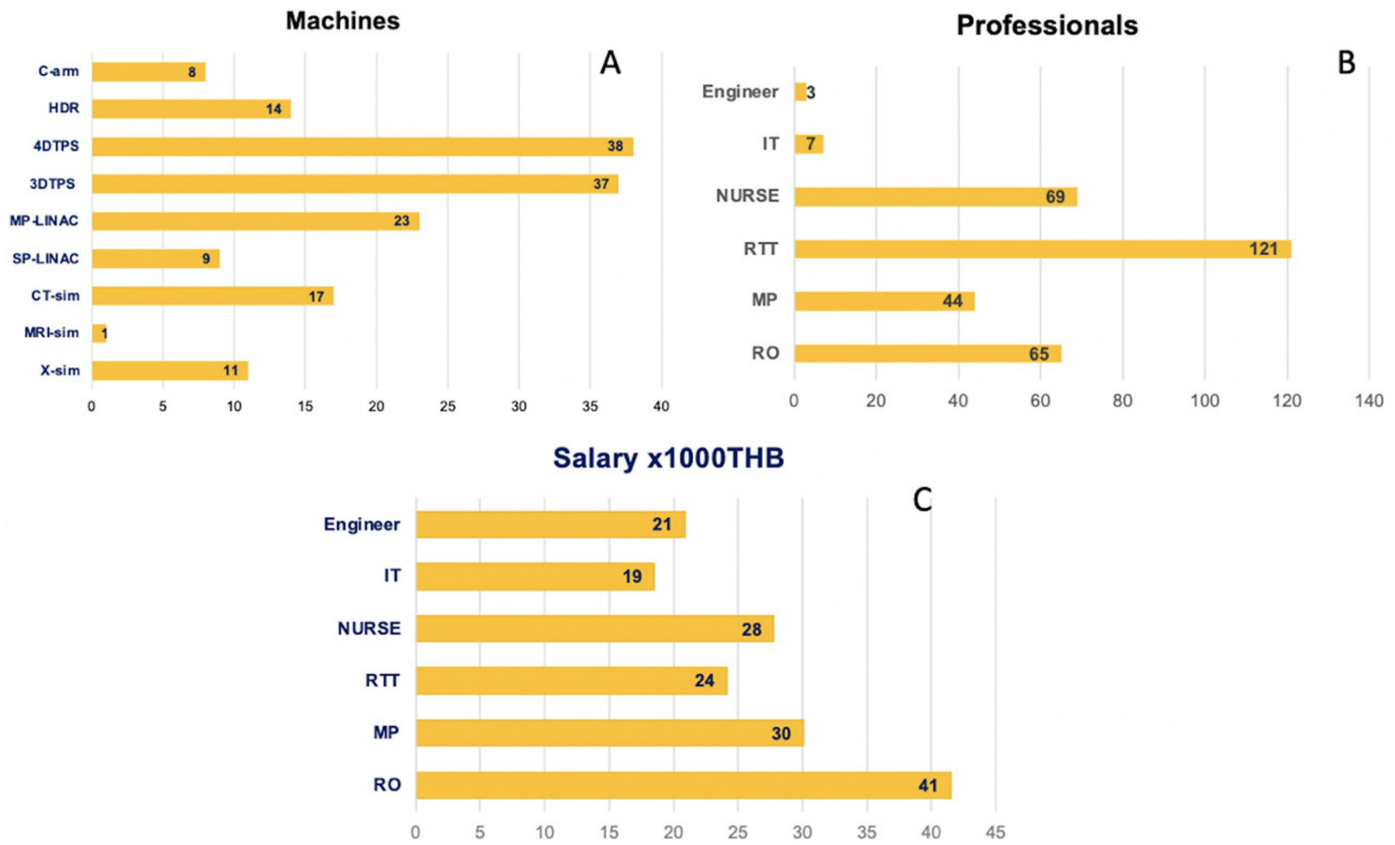
The quantities of **(A)** machines, **(B)** the number of professionals, and **(C)** monthly salaries. HDR, high-dose-rate remote afterloading machine; TPS, treatment planning system; MP-LINAC, multi photon linear accelerator; SP-LINAC, single photon linear accelerator; CT-sim, computed tomography simulator; MRI-sim, magnetic resonance imaging simulator; IT, information technology technician; X-sim, X-ray simulator; C-arm, C-arm fluoroscopy; RO, radiation oncologist; MP, medical physicist; RTT, radiation technologist.

Our facilities included 11 fluoroscope simulators, 17 CT simulators, 1 MRI simulator, 32 linear accelerators, 75 treatment planning workstations, 14 HDR machines, and 8C-arms. One CT scanner was specifically designated for brachytherapy. The “full-time” staff comprised 65 radiation oncologists (RO), 44 medical physicists (MP), and 121 radiation technologists (RTT). The average number of external beam radiotherapy fractions for gynecologic cancer treatments was 26. Figure 1 shows the details of the infrastructure characteristics.

From the 14 centers, a total of 18,024 patients were treated with RT, including 2,950 with gynecological cancers. Of these, 2,081 patients underwent a combination of external beam radiotherapy and brachytherapy, with 72% of this group diagnosed with cervical cancer.

In the case of cervical cancer patients, 30% were aged 51–60. Stage distribution showed group 3 represented stage (IB3-IVA in FIGO 2018) as the most prevalence.

In 2019, brachytherapy techniques across 14 RT centers were as follows: 4 centers used 2DBT techniques, 5 centers used IGBT techniques, and 5 centers employed a mix of 2DBT and IGBT techniques. To assess A-IGBTU, the number of fractions was selected since a mixed technique could be applied to the same patient. This survey recorded a total of 6,232 fractions, with 33% of these involving IGBT using CT or MRI. Details of brachytherapy utilization in cervical cancer are presented in Supplementary material.

### Step 1.2: the prediction of the requirement of RT for cervical cancer in 2040

**Figure 2:**
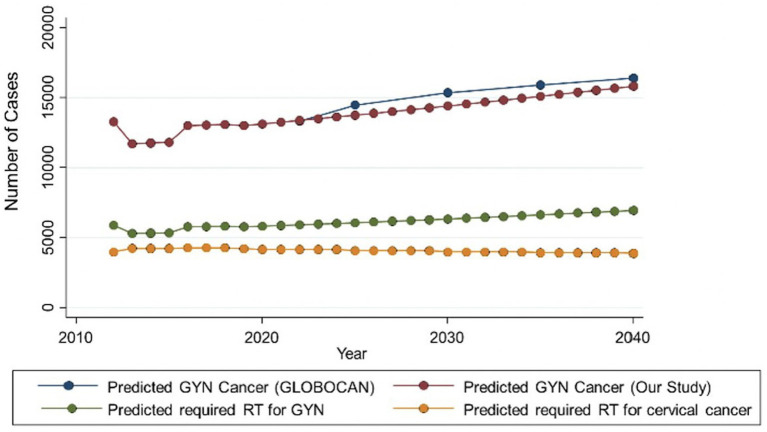
Prediction of required RT for cervical cancers in Thailand.

Using data from the THASTRO database, the RT requirements for cervical cancer shows a tendency to decrease from 2012 to 2040, whereas the RT requirement for GYN will increase slightly (Figure 2). The increasing requirement of RT for GYN is likely to be dependent on the increasing incidence of corpus cancer. From the THASTRO database, cervical cancer shows a projected tendency to decrease in the future based on the data available from 2012 to 2018. This may be due to the efficacy of the screening programs that have become widespread in Thailand since 2005.

### Step 2: shortage evaluation by the RRCC (version 21.0)

Using the RRCC (version 21.0), the evaluations revealed a shortfall of personnel relative to the workload. The available workload coverage for RO, MP, and RTT was 58, 37, and 39%, respectively. These findings highlight deficiencies in radiotherapy staff and linear accelerators as evidenced by our analysis. The calculation of equipment and staff requirements includes both EBRT and brachytherapy. The methodology is time-driven activity-based costing. The details on the current shortages and the ideal staffing levels needed to meet the workload were presented in Supplementary material.

**Figure 3:**
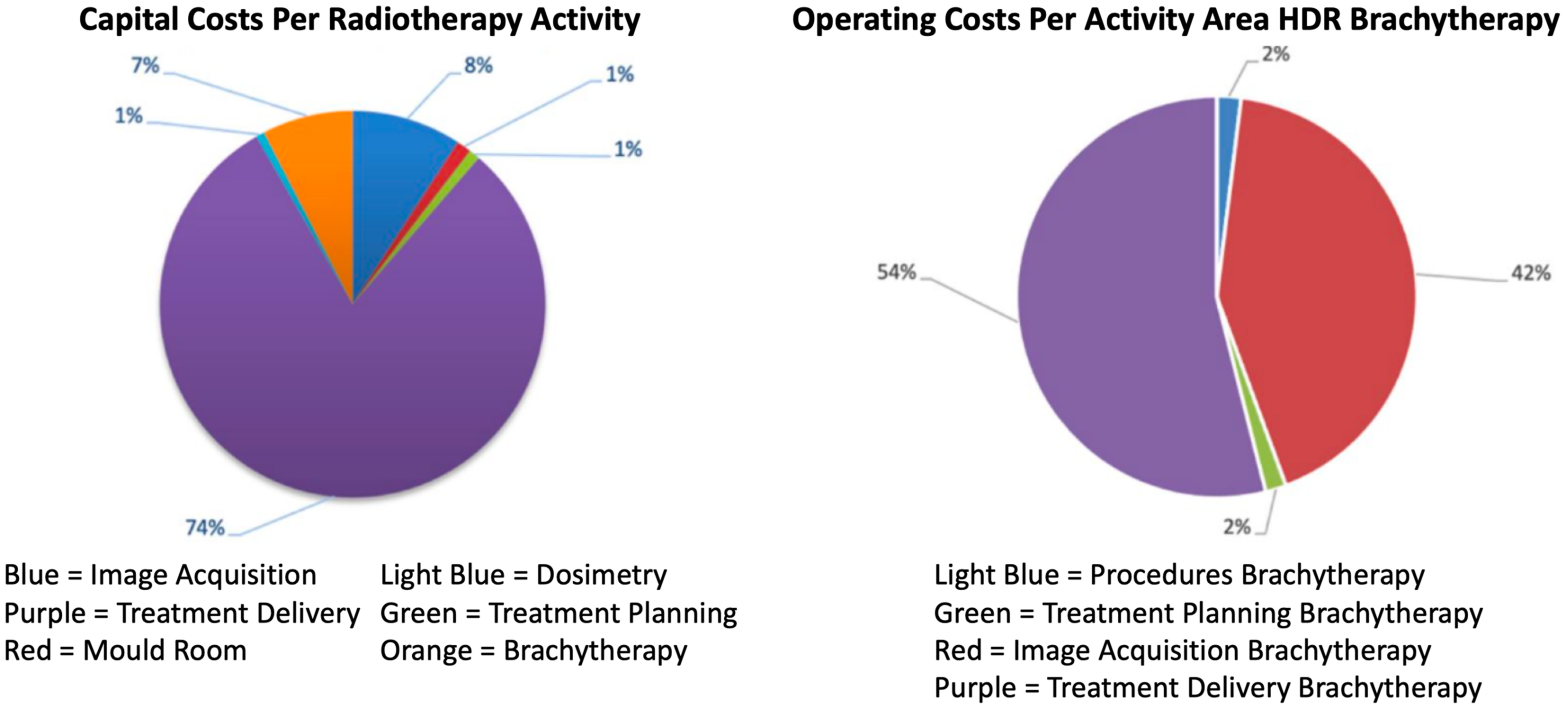
Percentages of capital costs and breakdown of operational costs for brachytherapy.

Regarding capital costs, brachytherapy accounted for 7% of the total capital costs per activity. For HDR brachytherapy, the highest cost was treatment delivery in brachytherapy, comprising 54%. Figure 3 illustrates the details of these costs.

### Step 3: development of an economic model

#### Step 3.1: the clinical outcome model

We analyzed our outcomes for 2DBT and IGBT within our institution. The treatment outcomes for 2DBT were reported by Lorvidhaya et al. in 2000, while the data for IGBT, reported by Tharavichitkul et al. in 2022, were used for calculations (13, 14). The results are presented in Supplementary material. Although the difference in overall survival (OS) rates between 2DBT and IGBT was roughly 1% (68.2% vs. 69%), differences were observed in advanced stages: a 4% increase in OS for stage IIB and a 10% increase in OS for stage IIIB.

To calculate the “person-life year” and ““total income of surviving patients,” we used the following data: (1) the average age of death in the Thai population, (2) the national income of Thai people per person, and (3) the survival outcome from Lorvidhaya et al. and Tharavichitkul et al. (13, 14). From our cohort, cervical cancer accounted for 72% of all gynecological cancers (2,124 patients), and 59% of cervical cancers (1,253) were group III (locally advanced cervical cancer patients who need combined chemoradiotherapy plus brachytherapy). The most common age group in our cohort of cervical cancer was 51–60 years (30%), and then the time to retirement was 10 years. In the Thai population, the average age at death was 78 years. According to the National Economic and Social Development Council in 2023 (17), the average annual income for the Thai population was 255,868 THB per person, and the age of retirement in Thailand was 60 years. Consequently, the “person-life year” was calculated from the survival rate × numbers of cervical cancer × time interval to death (from 50 to 78 years = 28 years) and the total income of surviving patients was calculated from the survival rate × numbers of cervical cancer × national annual income of the Thai population × 10 years (from 50 to 60 to retirement). The details of person-life years and total income of surviving patients are shown in Table 1. From our calculation, especially in the locally advanced group (IIB-IVA), which strongly need brachytherapy, IGBT yielded a difference in total income among surviving patients of ISD6 + million.

**Table 1:** The economic outcomes between 2DBT and IGBT.

Parameters	2DBT	IGBT	Difference
Person-life year in overall (years)	40,441	41,036	595
Person-life year in group III locally advanced cervical cancer (years)	21,401	23,857	2,456
Total income of all surviving patients (USD)	102,654,242	104,163,863	1,509,621
Total income of surviving patients in group III (Locally advanced cervical cancer) (USD)	54,324,330	60,558,270	6,233,940

USD1 = 36THB; the minimal time until retirement used 10 years; USD, United States Dollars; 2DBT, two-dimensional brachytherapy; IGBT, Image-Guided Brachytherapy.

#### Step 3.2 the toxicity model

A total of 625 episodes of toxicities were recorded between 2013 and 2023 in our institution. After 94 episodes were eliminated because our medical records did not contain all the necessary information, 531 episodes remained. Among these 395 episodes in 187 patients, 187 underwent brachytherapy; in our analysis, fistula was the most commonly noted toxicity, followed by proctitis. Table 2 shows the distribution of strategies by toxicity grade. While assessing each patient, 58 patients received three-dimensional brachytherapy (3DBT) treatment, 124 patients received 3DBT treatment, and 124 received 2DBT treatment. Our evaluation found that all instances of proctitis and cystitis were classified as grade-2 toxicity, while other complications such as hematochezia, hematuria, bowel obstruction, and fistula were categorized as grade-3 or grade-4 toxicity.

**Table 2:** Severe complications in 2DBT vs. IGBT.

Complications per episode	Grade 3	Grade 4
2DBT	41	120
IGBT	32	44
Complications per patient		
2DBT	21	54
3DBT	17	23

2DBT, two-dimensional brachytherapy; IGBT, Image-Guided Brachytherapy.

As shown in Table 2, patients with grades 3–4 side effects from two-dimensional brachytherapy (2DBT) experienced 161 episodes, while those undergoing three-dimensional brachytherapy (3DBT) experienced 76 episodes. Patients with grade 3–4 side effects required hospitalization for at least 3 days for evaluation and treatment. For example, if the DRG reimbursement was approximately 60,000 THB (approximately USD1,667) per 3-day admission to treat serious complications. Therefore, the total DRG reimbursement for 2DBT and 3DBT from 2013 to 2023 amounted to 9,660,000 THB (approximately USD268,333) and 4,560,000 THB (approximately USD126,667), respectively. Image-Guided Brachytherapy (IGBT) demonstrated a lower treatment cost for managing severe toxicity.

## Discussion

The practice and use of IGBT has begun and increased since 2000. The first two publications pertinent to the European Brachytherapy Group–European Society for Therapeutic Radiology and Oncology (GEC–ESTRO) recommendations were published in 2005 and 2006, followed by the first clinical impact of MRI-guided adaptive brachytherapy in 2007, with promising treatment outcomes (18–20). After this publication, many radiotherapy centers worldwide gained this knowledge, and IGBT was implemented.

In Thailand, the first publication on IGBT using computed tomography was published in 2011 by the team from the Faculty of Medicine, Chiang Mai University, and the results showed that IGBT could reduce unnecessary dose to the organs at risk (bladder, rectum, and sigmoid) (21). These results encouraged the team from CMU to transform to total IGBT use, a transition which was finally completed in 2019.

Optimal (o-BTU) and actual (a-BTU) brachytherapy utilization were first reported in 2006 by Thomson et al. from Australia, with o-BTU and a-BTU rates of 49 and 30%, respectively (22). However, no report about the utilization of IGBT was published at that time.

Until now, the latest report concerning the utilization of brachytherapy in cervical cancer was 76%, cited by Han et al. in 2024 (23). A recent American Brachytherapy Society (ABS) survey showed a 94% IGBT utilization rate (24).

Moreover, according to Image guided intensity modulated External beam radiochemotherapy and MRI-based adaptive Brachytherapy in locally advanced Cervical cancer: EMBRACE and ESGO/ESTRO/European Society of Pathology (ESP) guidelines in 2023, IGBT was recommended as the standard treatment in cervical cancer (7, 25). With these pieces of evidence, the optimal IGBT utilization rate in brachytherapy for patients with cervical cancer who need brachytherapy can be defined as 100%.

Our 2019 data showed that the actual IGBT utilization rate for cervical cancer across these 14 centers was 33%. The gap that we need to fill in is more than 60%. The main reason for the gap was the workforce. According to the RRCC version 21.0 analysis, the workforce numbers (RO, MP, and RTT) indicate a shortage. At least 40, 60, and 60% of ROs, MPs, and RTTs, respectively, were needed to fulfill the workload of the whole RT process. In the case of brachytherapy, the shortage is more likely due to the lack of Brachytherapy specialists in Thailand. Moreover, several challenges may hinder the full implementation of Image-Guided Brachytherapy (IGBT).

These include the time intensiveness of the IGBT procedure, insufficient specialized training programs in brachytherapy, restrictive reimbursement protocols, and the substantial capital investment required for additional imaging modalities such as magnetic resonance imaging (MRI) or Computed Tomography (CT) (26, 27).

As regards economic benefits, IGBT yielded greater gains. Chakraborthy et al. reported a significant difference in income generated among women treated with MRI-Guided Brachytherapy at Tata Memorial Hospital. Outcome data were simulated from 463 patients treated in their center. Based on their analysis, the cumulative income in 5 years ranged between ₹101–168 million if all patients were treated by MRI-Guided Brachytherapy (28).

Our study showed the same benefits. IGBT resulted in more patient-life years and a higher total income of surviving patients in comparison to 2DBT. From our cohort of 14 centers (around 1/3 of the total centers in Thailand), at least 588 years and USD1.5 million were gained when we changed to IGBT in all of our cervical cancer patients.

Regarding the toxicity profiles, the data analysis in MED-CMU showed promising results. IGBT showed a 50% decrease in the incidence of grade 4 toxicity compared with 2DBT in our cohort, from the data collected from 2013 to 2003. Additionally, our analysis revealed that the total DRG reimbursement for treating serious complications with IGBT was less than half of that for 2DBT.

Our analysis has some limitations. First, we only evaluated the financial impact of IGBT in the management of cervical cancer in Thailand. However, our study is not a pure health economic study. Second, this analysis was conducted in 14 centers across Thailand. Third, due to the retrospective approach, data in some areas were missing due to manual recording, which could not be checked. Fourth, some data on survival increases and toxicity were collected from a single center, limiting transferability. Fifth, we used data from 2019—a period with minimal disruption from the coronavirus disease 2019 (COVID-19) pandemic—now considered outdated in light of current clinical and economic conditions. Sixth, a direct comparison of the two datasets using identical parameters (e.g., 5-year survival) was not feasible because the median follow-up time for the second cohort was only 48 months compared with 96 months in our previous publication (13, 14).

However, our analysis showed the status of the actual IGBTU in our country, the gap we need to fill, the return on investment (ROI) based on survival-life years and total income, and the reduced incidence of toxicity. Complete transfer to the use of IGBT would benefit our cervical cancer patients, and its use needs to be encouraged in routine practice. To further optimize the nationwide utilization of IGBT, policy reforms are essential. In particular, improved resource allocation, the development of enhanced training programs, and revisions to reimbursement policies are strongly recommended to facilitate broader adoption and sustained use of IGBT within the national healthcare system.

The nationwide prevention strategy for cervical control has been implemented in Thailand since 2005 for national screening under universal health care (UHC) (29) and in 2018 for human papillomavirus (HPV) vaccination in young women aged 11 years (30). However, the data pertinent to the coverage of screening and vaccination for the prevention of cervical cancer, which may influence the decrease in the trend of cervical cancer incidence, were not considered in the prediction because of the limited data. This would be an interesting avenue to pursue in future studies.

## Data Availability

The raw data supporting the conclusions of this article will be made available by the authors, without undue reservation.

## Footnotes

1 https://www.thastro.org/
