# Supplementary material for: Unlocking economic gains: the impact of image-guided brachytherapy on cervical cancer treatment in Thailand
Source: Front Public Health. 2026 Feb 2;13:1725415. doi: 10.3389/fpubh.2025.1725415 (PMC12907340; doi:10.3389/fpubh.2025.1725415)
Supplement: Supplementary file 1 [file Supplementary_file_1.docx]

**Supplementary Figure 1** link of web-based questionnaires to 14 centers in Thailand

<https://tinyurl.com/nbmktzjb>

<https://jokekittikun.github.io/RCA-Thailand>

**Supplementary Figure 2** The study procedure

**Supplementary Figure 3** The actual IGBT utilization rate for cervical cancer.


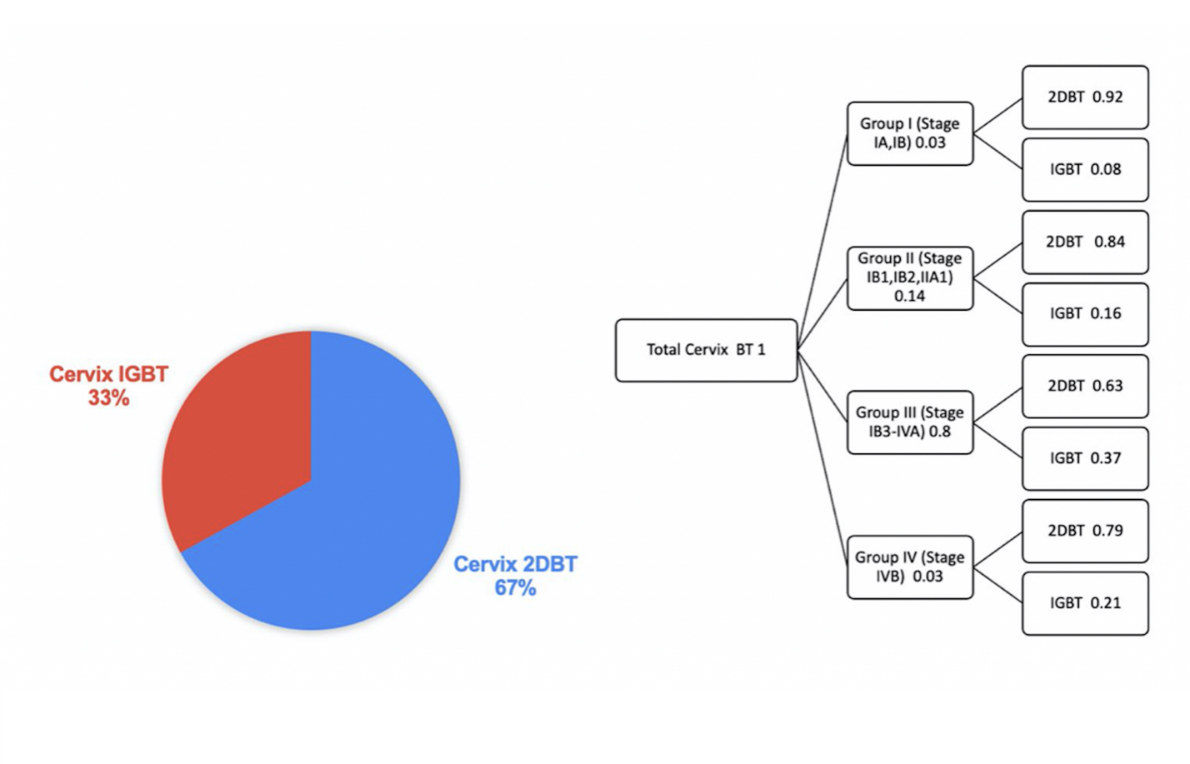


**Supplementary Figure 4** Available percentage of A) radiotherapy staffs, B) recommended staffs and C) available/needed hours of machines

**
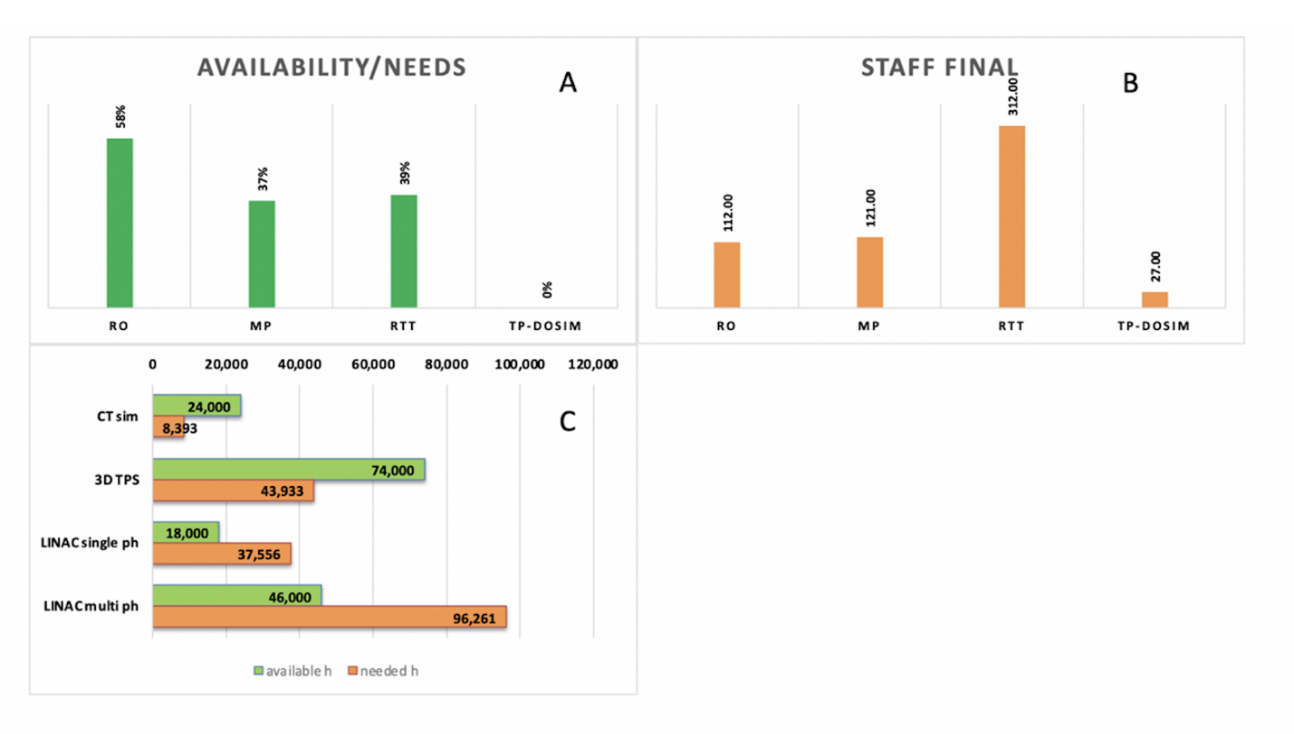
**

**Supplementary Table 1** Comparison of data between 2D-BT and IGBT used for calculations

| Parameters | Lorvidhaya, et al. 2000[11] | Tharavichitkul, et al. 2022[12] |
| --- | --- | --- |
| Type of BT | 2D-BT (point A) | IGBT (CT or TAUS) |
| Number of patients | 1992 | 295 |
| Median follow-up time (months) | 96 | 48 |
| Local control rate (All stages) | 79.2% (5-yr) | 89%(4-yr) |
| Overall survival rate (All stages) | 68.2% (5-yr) | 69.1% (4-yr) |
| Overall survival rate of group III (Locally advanced Cervical cancer) | 61% (5-yr) | 68% (4-yr) |

Note: 2D-BT=two-dimensional brachytherapy; IGBT = image-guided brachytherapy; yr=year; CT=computed tomography, TAUS=transabdominal ultrasound
